# Poly(furfuryl alcohol)-Polycaprolactone Blends

**DOI:** 10.3390/polym11061069

**Published:** 2019-06-20

**Authors:** Gabriele Nanni, José A. Heredia-Guerrero, Uttam C. Paul, Silvia Dante, Gianvito Caputo, Claudio Canale, Athanassia Athanassiou, Despina Fragouli, Ilker S. Bayer

**Affiliations:** 1 Smart Materials, Istituto Italiano di Tecnologia, via Morego 30, 16163 Genova, Italy; Jose.Heredia-Guerrero@iit.it (J.A.H.-G.); Uttam.Paul@iit.it (U.C.P.); Athanassia.Athanassiou@iit.it (A.A.); Despina.Fragouli@iit.it (D.F.); 2 Nanoscopy & Nikon Imaging Center, Istituto Italiano di Tecnologia, Via Morego 30, 16163 Genova, Italy; Silvia.Dante@iit.it; 3 Nanochemistry, Istituto Italiano di Tecnologia, Via Morego 30, 16163 Genova, Italy; Gianvito.Caputo@iit.it; 4 Department of Physics, Università degli studi di Genova, 16146 Genova, Italy; canale@fisica.unige.it

**Keywords:** poly(furfuryl alcohol), polycaprolactone, biocompatibility, antioxidant polymer, food packaging

## Abstract

Poly(furfuryl alcohol) (PFA) is a bioresin synthesized from furfuryl alcohol (FA) that is derived from renewable saccharide-rich biomass. In this study, we compounded this bioresin with polycaprolactone (PCL) for the first time, introducing new functional polymer blends. Although PCL is biodegradable, its production relies on petroleum precursors such as cyclohexanone oils. With the method proposed herein, this dependence on petroleum-derived precursors/monomers is reduced by using PFA without significantly modifying some important properties of the PCL. Polymer blend films were produced by simple solvent casting. The blends were characterized in terms of surface topography by atomic force microscopy (AFM), chemical interactions between PCL and PFA by attenuated total reflection-Fourier transform infrared (ATR-FTIR), crystallinity by XRD, thermal properties by differential scanning calorimetry (DSC), and mechanical properties by tensile tests and biocompatibility by direct and indirect toxicity tests. PFA was found to improve the gas barrier properties of PCL without compromising its mechanical properties, and it demonstrated sustained antioxidant effect with excellent biocompatibility. Our results indicate that these new blends can be potentially used in diverse applications ranging from food packing to biomedical devices.

## 1. Introduction

Furfural has been identified as a very important and promising chemical precursor directly derived from biomass. Furfural annual production is approximately 300 kTon and its production and purification technologies are well established today [1]. Furfural is produced from hydrolysis of pentosane-rich, abundant biomass such as corncob and sugar cane bagasse, rice hulls, olive husks and woodchips [2]. As such, any resin or polymeric compound derived from furfural is regarded as petrochemical-free, biomass origin resin or compound. 

Most of the furfural produced worldwide is converted by hydrogenation into furfuryl alcohol (FA) [3], which can be readily polymerized through cationic condensation to obtain polyfurfuryl alcohol (PFA). Due to its excellent thermal stability, remarkable resistance to acidic conditions, as well as to fire and corrosion induced degradation, PFA is largely used in the formulation of wood adhesives [4], as binding agent in foundry sand [5], and in corrosion-resistant glass fiber-reinforced composites. Notably, PFA gives a high carbon yield if undergoes pyrolysis [6]. Therefore, PFA is also the precursor choice for fabricating nanostructured carbon materials and carbon-based nanocomposites for applications such as molecular sieve adsorbents and electrodes [7,8,9,10]. FA can be also polymerized in situ in the presence of other polymers to create special membranes that resist acidic or basic media [11] or for fuel cell applications [12].

In composite science and technology, PFA has been used in the design of green composites originating from natural fibers like kenaf, with a significant improvement of the mechanical properties such as tensile, flexural, impact strength and storage modulus [13]. Toriz et al. [14] proved that adding PFA to cellulose ester flax fiber composites results in the enhancement of the modulus and rupture strength of these biocomposites. Guigo et al. [15] fabricated PFA/lignin thermosetting green composites having good thermo-mechanical performance with no phase separation. In another work, incorporation of lignin in PFA showed significant improvements in flexural strength, elastic modulus, and storage modulus over pure PFA [16]. Pranger et al. [17] employed an in situ polymerization approach to produce PFA nanocomposites with cellulose nanowhiskers and nanoclay. Both bio-nanocomposites were characterized by significantly higher temperature at the onset of degradation and higher residual weight after non-oxidative degradation compared to unmodified PFA resins. However, composites or blends of PFA with other biodegradable thermoplastics are rather scarce. Sharib et al. [18] dissolved poly lactic acid (PLA) in FA to form a thermosetting PFA resin containing PLA. They investigated whether or not the curing time could be decreased in the presence of acidic groups of PLA. The second objective was to optimize PLA concentrations in PFA (0.5% to 3.0% *w*/*v*) to achieve higher elongation at break values. The fabricated PFA-based bioplastics were subjected to mechanical, thermal and biodegradability studies. 

Polycaprolactone (PCL) is a synthetic biodegradable polyester with a low melting point of around 60 °C. PCL is a popular oil-derived biodegradable thermoplastic and has several uses in different fields such as scaffolds in tissue engineering [19,20,21,22], drug delivery [23], and packaging [24]. PCL is known to be compatible with many other bio-based thermoplastics and petroleum-based polymers as well as polyurethanes [25]. To the best of our knowledge, blends of PCL with other thermosetting resinous materials generally focus on PCL-epoxy systems [26] in which PCL acts as a mechanical property modifier and helps electrospinning of epoxies for functional applications [27]. Herein, we investigate PCL-PFA blends for the first time. The combination of a biodegradable polymer with a biomass-derived resin is expected to generate new biomaterials with attractive novel properties. We combined PCL and PFA in different proportions and investigated the compatibility of the two phases with atomic force microscopy (AFM). Moreover, biocompatibility of the blends was also assessed. Due to PFA, the blends demonstrated good antioxidant properties as well as low oxygen permeability without significantly altering the appealing mechanical properties of PCL for many applications.

## 2. Materials and Methods 

### 2.1. Materials

Polyfurfuryl alcohol was purchased from Polysciences Inc. and used as received. Polycaprolactone pellets (average *M*_n_ 80,000), reagent grade dichloromethane (DCM) and ethanol were purchased from Sigma-Aldrich (Buchs, Switzerland) and used as received. Dulbecco’s modified eagle medium (DMEM), phosphate buffer saline (PBS), glucose, glutamine, fetal bovine serum inactivated, penicillin streptomycin and non-essential amino acids used for cell culture were produced by Gibco (Dublin, Ireland) and used as received. Hoechst and Alexa-Fluor 488-phalloidin for cell staining were purchased from Thermo Fisher Scientific (Waltham, MA, USA).

### 2.2. Methods

#### 2.2.1. Film Preparation 

The films were prepared by separately dissolving PCL and PFA resins in DCM (both at 100 mg/mL) and subsequent solution mixing using various PCL/PFA proportions (95/5, 90/10, 70/30, 50/50 wt %). The solutions were stirred thoroughly at room temperature and then gently poured in glass Petri dishes, after which the solvent was allowed to evaporate gently under a standard aspiration hood overnight. After solvent evaporation, the obtained films did not adhere to the glass Petri dishes and were easily peeled off.

#### 2.2.2. Atomic Force Microscopy (AFM Characterization) 

AFM experiments were performed using a Nanowizard III system (JPK Instruments, Germany) mounted on an Axio Observer D1 inverted optical microscope (Carl Zeiss, Oberkochen, Germany). The images were acquired in tapping mode in air at room temperature. Single beam silicon cantilevers (TESPA, NanoAndMore USA Corp., Watsonville, CA, USA) with a nominal spring constant of 42 N/m and resonance frequency of 320 kHz were employed. Images with two different scan sizes (2 × 2 μm^2^ and 10 × 10 μm^2^) were acquired on five different areas on each sample. The scan rate was between 0.5 Hz and 1.0 Hz.

#### 2.2.3. Attenuated Total Reflection-Fourier Transform Infrared (ATR-FTIR) Characterization

Infrared spectra were obtained with an ATR accessory (MIRacle ATR, PIKE Technologies, Fitchburg, WI, USA) coupled with a FTIR spectrometer (Equinox 70 FT-IR, Bruker Optik, Ettlingen, Germany). All spectra were recorded in the range from 3800 to 600 cm^−1^ with a resolution of 4 cm^−1^, accumulating 128 scans. To assess the homogeneity of chemical composition, ATR-FTIR spectra were recorded three times in different areas.

#### 2.2.4. Thermal Analysis

Differential scanning calorimetry (DSC) thermograms were acquired with a DSC Q20 (TA Instruments, New Castle, DE, USA) from 25 °C to 100 °C under dry nitrogen flow (50 mL/min) at 10 °C/min. Accurately weighed small pieces (~4 mg) were cut from the films and stabilized at 55% relative humidity (RH) in a glove box for 5 days. After stabilization, samples were hermetically closed in aluminum pans inside the glove box and a pinhole was made immediately before running the DSC experiment. A heating-cooling-heating cycle was performed. The melting point and the enthalpy of fusion were obtained from the second heating.

The thermal degradation behavior of the films was investigated by a standard thermogravimetric analysis (TGA) method using a TGA Q500 by TA Instruments (New Castle, DE, USA). Measurements were performed on 3–5 mg samples in an aluminum pan under inert N_2_ atmosphere with a flow rate of 50 mL/min in a temperature range from 30 to 800 °C with a heating rate of 10 °C/min. The weight loss and its first derivative were recorded simultaneously as a function of time/temperature.

#### 2.2.5. Determination of Sample Crystallinity

X-ray diffraction (XRD) measurements were performed on a PANalytical Empyrean X-ray diffractometer using a Cu Kα anode (*λ* = 1.5406 Å) operating at 45 kV and 40 mA. The diffraction patterns were collected in the range 10°–70° 2θ with a 0.03° step size. Samples crystallinity (*χ*_c_) was evaluated according to the following relationship:

*χ*_c_ (%) = [*A*_c_/(*A*_c_ + *A*_a_)] × 100 (1)

where *A*_c_ and *A*_a_ correspond to the area related to crystallized and amorphous components, respectively.

#### 2.2.6. Mechanical Analysis

The Young’s modulus of the blends was experimentally determined by uniaxial tensile tests using an Instron 3365 machine. Tensile testing was conducted at environmentally controlled conditions (20 °C, 40% RH). Specimens for the tensile test were obtained by using a punching machine. Dog-bone shape specimens were created using a hollow die with a gage length of 25 mm and the width of the reduced section of 3.98 mm. To collect stress-strain curves, the samples were stretched at a rate of 5 mm/min. Five specimens for each material were tested, and the results were averaged to obtain a mean value and the standard deviation.

#### 2.2.7. Biocompatibility Study 

Chinese hamster ovarian cells (CHO) (ATCCs, UK) were cultured in Dulbecco’s modified eagle medium (DMEM). DMEM is the most commonly utilized medium for many adherent cell phenotypes among defined media for cell and tissue culture. The Dulbecco’s modification is an enhanced supplementary formulation that boosts select amino acid and vitamin content of the original eagle’s medium by up to fourfold. DMEM generally contains containing 4.5% glucose and glutamine, 10% fetal bovine serum inactivated, 1% penicillin streptomycin and 1% non-essential amino acids in an incubator at 37 °C and 5% CO_2_.

For direct biocompatibility tests, PCL/PFA films were first sterilized under UV for 30 min, rinsed with sterile water. The sterile PCL/PFA films were submerged in DMEM culture medium for 48 h, to remove any possible potentially toxic leftover solvents or contaminants. The medium was then exchanged with fresh DMEM, and CHO cells were seeded on the substrates at a density of 2 × 10^4^ cells/mL and let to grow in the incubator. CHO cells were kept in culture for 48 h, until they were confluent. Cells were fixed in a 4% formaldehyde solution in PBS for 10 min at room temperature. CHO staining was carried out as reported elsewhere in detail [28]. Shortly, CHO nuclei were stained with Hoechst (Thermo Fisher Scientific, Waltham, MA, USA), 1 μg/mL for 20 min Alexa-Fluor 488-phalloidin was used to label the cytoskeleton. The PCL/PFA films were mounted on glass coverslips using ProLong mounting medium. The cells were imaged using a Nikon Inverted Microscope TiE equipped with a Nikon Confocal Laser System (Nikon Optical Co., Ltd., Tokyo, Japan) at an excitation wavelength *λ*_ex_ = 405 nm, and *λ*_ex_ = 488 nm at a 20× magnification. 

The indirect toxicity test was conducted by a proliferation assay in the culture medium previously conditioned in the presence of PCL/PFA films for 48 h. The cell proliferation assay was performed with an xCELLigence device (ACEA Biosciences) equipped with E-plate 16 with a view window for optical inspection. The device allows the real-time monitoring of cell viability, based on electrical impedance read out. Each of the 16 well was filled with 100 μL of conditioned DMEM. After background measurement, 100 μL (1000 cells/μL) of CHO cell suspension in DMEM was added to the wells. For the control samples 100 μL fresh DMEM were added to the wells. 

The device was kept into the incubator and left to run for 7 days. Sampling of the cell proliferation was done every 30 min by reading out the impedance of the electrodes, and converting it to a dimensionless parameter, named cell index (CI) proportional to the electrode area covered by the cells [29]. The CI matrix was then processed by Microcal’s Origin.

#### 2.2.8. Determination of Antioxidant Activity

The free radical scavenging activity of films of PCL and PCL/PFA blends was determined with 2,2-diphenyl-1-picrylhydrazyl (DPPH•) free radical scavenging assay. 0.004 g of DPPH was dissolved in 100 mL of ethanol to generate a 0.1 mM solution. Working solutions were prepared fresh before each experiment. 3mL of diluted DPPH• were pipetted into a polystyrene cuvette into which a small circular piece of sample (diameter 8 mm, mass ~13 mg) was previously introduced. The cuvettes were closed with a cap as soon as the diluted DPPH• was pipetted. The absorbance of the solutions containing the films was hourly measured in an automated way for 24 h using a UV–visible spectrophotometer (Cary 6000i from Varian). The capability of scavenging the radical DPPH• was calculated by using the following formula:

*Scavenging activity* (%) = [1 − *A*_sample_/*A*_control_] × 100 (2)

where *A*_control_ is the absorbance maximum centered at a wavelength of 517–526 nm of the control (ethanol solution of DPPH•), and *A*_sample_ is the absorbance maximum centered at a wavelength of 517–526 nm for the ethanolic solution of DPPH• containing the film.

#### 2.2.9. Oxygen Transmission Rate Measurements

The oxygen permeation test of the PCL/PFA blend films were performed using an Oxysense 5250i device (Oxysense, New Castle, DE, USA) equipped with a film permeation chamber. This machine was operated according to ASTM Method F 3136–15 (ASTM, 1989). The test was performed at room conditions, i.e., 23 °C and 50% RH. The permeation chamber consists of a cylinder divided in two parts (sensing well and driving well). The sensing well is equipped with a fluorescence sensor called oxydots, sensitive to the oxygen. This chamber is purged with nitrogen while the other one (driving well) is kept open to ambient air. The circular film of 6 cm diameter was placed inside the chamber, which was properly closed. The OxySense fibre optic pen measures the oxygen reading from the oxydots, at specific time intervals. The oxygen transmission rate (OTR) of blend films was then measured by monitoring the oxygen uptake with time. Oxysense OTR software uses this oxygen evolution to determine the OTR of films. At least ten readings were taken for each sample with a minimum coefficient of determination value (*R*^2^) of 0.995.

## 3. Results

### 3.1. PCL/PFA Films Morphology and Surface Topography

**Figure 1:**
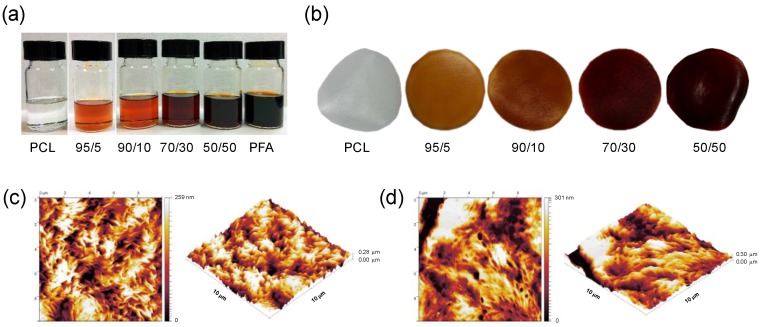
(**a**) Photographs of polymer solutions of polycaprolactone (PCL) and poly(furfuryl alcohol) (PFA) resin dissolved in dichloromethane (DCM) containing different PFA concentrations; (**b**) photographs of the blend films obtained after casting the solutions and drying. The films have a thickness of approximately 100 μm and a diameter of 3 cm. Atomic force microscopy (AFM) images and 3D rendering of (**c**) a PCL film and (**d**) a 50/50 blend film. The AFM images are 10 μm × 10 μm in size.

Free standing films can be easily obtained upon solvent mixing and casting into glass dish molds. PCL and PFA were both dissolved in DCM and mixed together in different proportions, as shown in Figure 1a. The amount of PFA ranged from 5 to 50 % of the total weight of the dry films. After drying, uniform dark red-colored films were obtained, as displayed in Figure 1b. No macroscopic phase separation was noticed. Surface topography was characterized by AFM, as shown in Figure 1c–d. AFM topographies revealed that PCL films and 50/50 blends (highest PFA concentration used) had a surface roughness of 50.59 ± 6.73 nm and 59.32 ± 15.38 nm, respectively. The fact that the surface topography of the 50/50 blend film was almost identical to pure PCL indicated that the two compounds were compatible, showing no phase segregation. The same surface topography observations were also made with other blends, i.e., 70/30. It is worth noting that blends can be subsequently melt processed by conventional means such as extrusion. Figure S1a shows representative extruded filaments of a 50/50 blend having different diameters obtained by extrusion using a bench top extruder (Figure S1b).

### 3.2. Chemical Analysis

**Figure 2:**
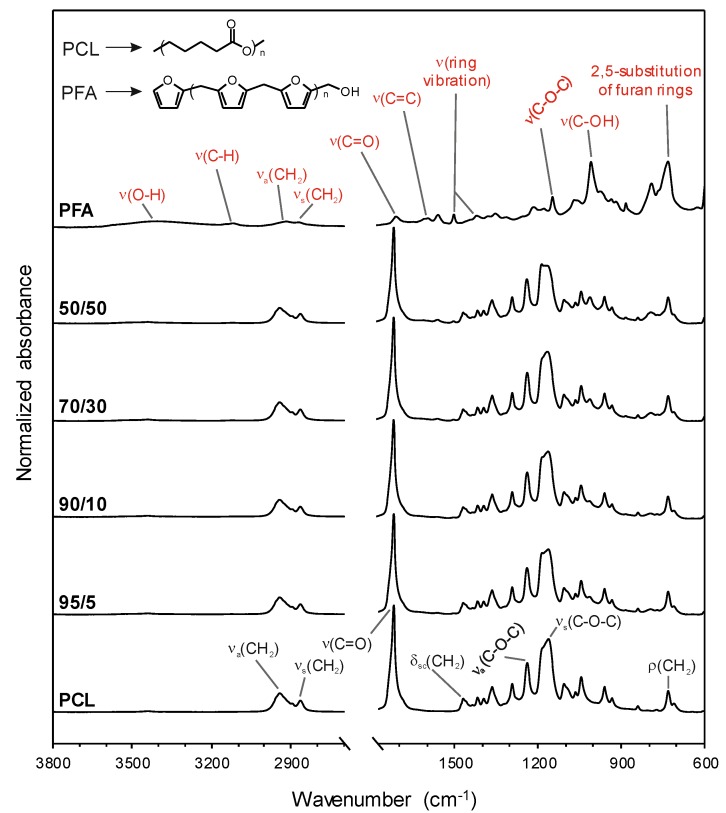
Attenuated total reflection-Fourier transform infrared (ATR-FTIR) spectra of pure PCL and PFA resin as well as blend films. The band assignments of PCL (black) and PFA (red) as well as the chemical structures of both substances are included.

The chemical characterization of pure PCL and PFA resins and blend samples was carried out by ATR-FTIR, Figure 2. For neat PCL, main bands were ascribed to methylene (mainly asymmetric and symmetric CH_2_ stretching modes at 2945 and 2866 cm^−1^, respectively, CH_2_ scissoring mode at 1470 cm^−1^ and CH_2_ rocking mode at 729 cm^−1^) and ester (C=O stretching mode at 1721 cm^−1^, and asymmetric and symmetric C−O−C stretching modes at 1240 and 1163 cm^−1^, respectively) functional groups [30,31]. On the other hand, for neat PFA, the assignment was as follows: OH stretching mode at 3406 cm^−1^, CH stretching mode in aromatic rings at 3119 cm^−1^, asymmetric and symmetric CH_2_ stretching modes at 2918 and 2869 cm^−1^, respectively, C=O stretching mode from open furan rings at 1713 cm^−1^, C=C stretching mode at 1604 cm^−1^, ring vibrations at 1564 and 1504 cm^−1^, C−O−C stretching mode at 1147 cm^−1^, C−OH stretching mode at 1009 cm^−1^, and a characteristic absorption of 2,5-substitution of furan rings at 733 cm^−1^ [7,32]. In the blend films, main absorptions were related to PCL, while some small bands (e.g., ring vibration at 1564 cm^−1^ and C−OH stretching mode at 1009 cm^−1^) could be ascribed to PFA. The absence of frequency shifts and intensity modifications suggested that blending of PFA and PCL did not form a new compound. Therefore, PCL and PFA formed polymer blends with no chemical interactions that exhibited macroscopically uniform physical properties.

### 3.3. Thermal Properties and Crystallinity

**Figure 3:**
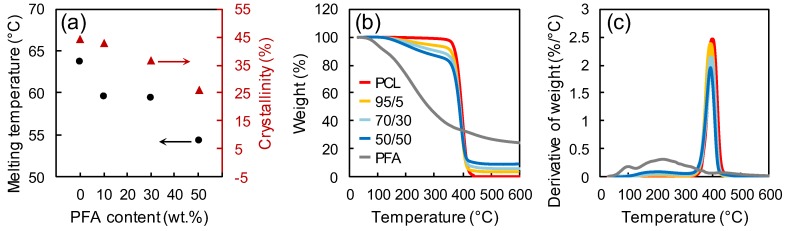
(**a**) Melting temperature and percent crystallinity for PCL and different PCL/PFA blends measured by DSC and XRD, respectively. Mass loss curves (**b**) and derivatives of each curve (**c**) for PFA, PCL and different PCL/PFA blends measured by TGA.

The investigation of blends’ thermal properties was helpful to determine the processing parameters of the blends. Figure 3a displays the melting temperature and the percent crystallinity of neat PCL and different PCL/PFA blends measured by DSC and XRD, respectively (XRD patterns are separately reported in Figure S2, Supporting Information). The presence of PFA had a significant effect on the thermal properties of PCL. The melting temperature for blends with 50 wt % of PFA was reduced by 9.3 °C compared to pure PCL. The decrease of the melting temperature could be directly related to the crystallinity loss of PCL induced by the presence of PFA [33,34]. Figure 3b,c show the mass loss curves and the derivatives of each curve of the samples, respectively. Neat PFA displayed two major mass losses. The first mass loss at ~100 °C was due to the sample dehydration, while chain scission and decomposition started above 200 °C [35]. The start of major mass loss in PCL samples occurred at ~400 °C, probably via an unzipping mechanism [36]. In PCL/PFA blends, two different mass loss steps at ~200 and ~400 °C accounted for the degradation of PFA and PCL, respectively. 

**Scheme 1:**
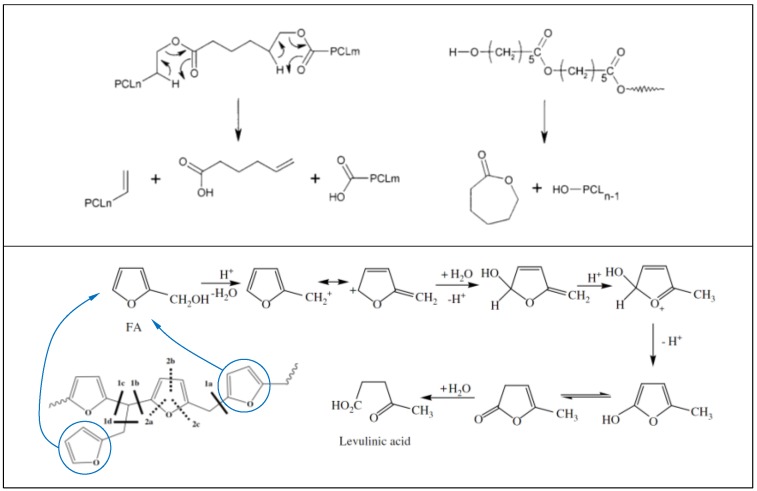
Thermal degradation mechanisms of PCL (top panel). Reproduced with permission from American Chemical Society [36]. Thermal degradation mechanisms of PFA (bottom panel). Reproduced with permission from Elsevier [35].

More specifically, PCL is known to degrade via double mechanism occurring at different temperatures [36]. The first degradation step generates water, CO_2_, and a carboxylic acid as evolved products. In PCL, pyrolysis results in chain cleavages that are randomly distributed all along the chain. When two pyrolysis reactions occur with neighboring ester functions, one of the reaction products is a volatile 5-hexenoic acid. CO_2_ forms by decarboxylation of 5-hexenoic acid or other carboxylic acid end groups (see Scheme 1, top panel). Evolution of water during thermal de-polymerization occurs by condensation reactions of hydroxyl and/or carboxylic acid functions in situ formed at high temperatures [36]. The evolution of ε-caprolactone monomer generally occurs around 430 °C, confirming our results and PCL chains depolymerize via an unzipping mechanism as schematized in Scheme 1 (top panel) below.

For PFA resin, it is known that after 350 °C, 2-methylfuran and 2-furfuryl-5-methylfuran are released with no furan ring opening reactions [35]. The unzipping of furan end groups by scission of methylene and methyne linkages leads to alkylfuranic volatile matter. These main scissions are referenced as scission 1 in Scheme 1 (bottom panel). The scission of 1d bond lead to 2-methylfuran. New ketonic volatiles appeared such as acetone, 2-butanone or 2-pentanone that can be formed by scission of the furan ring (called scission 2 in Scheme 1) together with continuation of methylene scission. For instance, since the C–O bond energy was lower than that of C=C, the furanic rings were primarily cleaved into enol type structure which lead to ketonic volatiles. Acetone was the major ketonic compound which was formed by scissions of 1a, 2a and 2b bonds (see Scheme 1 bottom panel). Formation of 2-butanone was attributed both to 1a, 2a and 2b scissions, while the 2-pentanone can be formed by 1a, 2a and 1b scissions shown in Scheme 1 [35]. The rate of this mechanism is strongly dependent upon the environmental conditions, particularly, water and moisture contents. 

### 3.4. Mechanical Properties

**Figure 4:**
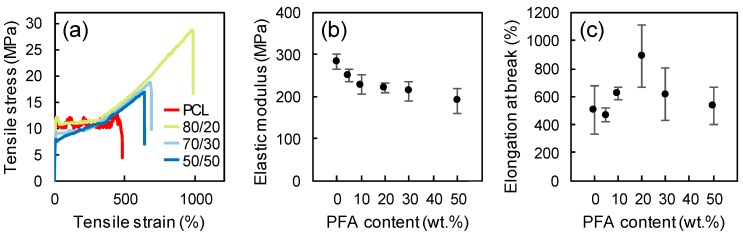
Representative stress-strain curves (**a**), elastic modulus (**b**), and elongation at break (**c**) of neat PCL and blends at different PCL/PFA proportions.

Figure 4 displays representative stress-strain curves, the elastic modulus and the elongation at break of neat PCL films and PCL/PFA films with different PFA concentrations. Neat PCL exhibited an elastic modulus of ~273 MPa. The addition of PFA gave rise to a decrease in the elastic modulus, while the elongation at break initially increased and tended to drop at PFA concentrations exceeding 20 wt %. Such results indicated that PFA at concentrations below 20 wt % acted as a plasticizing agent since it provided plasticity through physical mixing, and it was not chemically bonded to PCL [37]. The decline in elastic modulus was not significant even for the 50/50 blend film (around 30%), considering major suppression of elastic modulus of polymers due to standard plasticizers. For instance, the elastic modulus of PLA resins was reduced by almost 6 times at 20% plasticizer levels [38]. Moreover, the fact that even the blend obtained by replacing 50 wt % of PCL still behaved similarly to the pure PCL in terms of elongation at break was encouraging for the production of new materials integrating a biomass origin resin.

### 3.5. Biocompatibility Study

**Figure 5:**
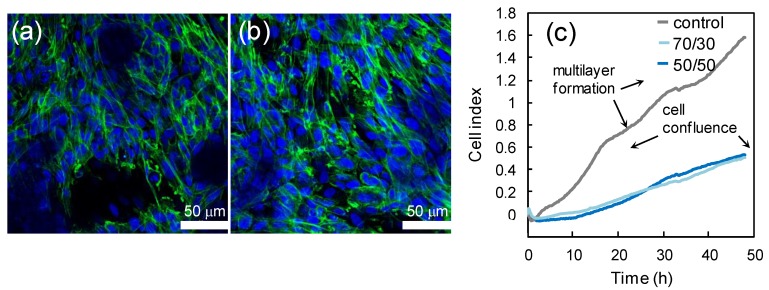
Confocal images of CHO cells growing on 50/50 PCL/PFA (**a**) and 70/30 PCL/PFA (**b**). Nuclei are labeled with DAPI (4′,6-Diamidino-2-phenylindole dihydrochloride, blue) and cytoskeleton is labeled with Alexa-Fluor Phalloidin (green). The cells morphology is healthy. The real-time proliferation curves are displayed in panel (**c**). On PCL/PFA, cells reached confluence after 48 h. The growth rate decreased compared to control, where cells built multilayers (see Figure S2), as the multistep curve indicates.

The biocompatibility of PCL/PCF films was tested seeding CHO cells onto films with different PFA/PCL proportions. Figure 5a,b display CHO cells cultured on 50/50 PCL/PFA and 70/30 PCL/PFA films, respectively. The cell morphology was regular, and cells reached confluence in all investigated samples, demonstrating the excellent biocompatibility of the substrates. No significant differences were observed between the different compositions of the films. The indirect toxicity test was carried out via a proliferation assay, measuring the CI as described in the Methods Section. The cell proliferation phase was characterized by a rapid growth of the CI and, when cells cover the entire available surface, the CI reaches the first plateau, Figure 5c. CHO seeded in fresh DMEM (control) were confluent in 24 h. The subsequent CI increase indicated that CHO cells build multilayers, as documented by the final optical images of the corresponding plate wells (Figure S3, Supporting Information). Instead, the cells seeded in the conditioned medium proliferate at a slower rate. Nevertheless, they are confluent after 48 h, for both 70/30 and 50/50 samples (see also Figure S3, Supporting Information). Cell survival and proliferation are allowed in the culture medium conditioned in the materials. Therefore, the toxicity of chemical agents released form the film is low.

### 3.6. Antioxidant Properties 

**Figure 6:**
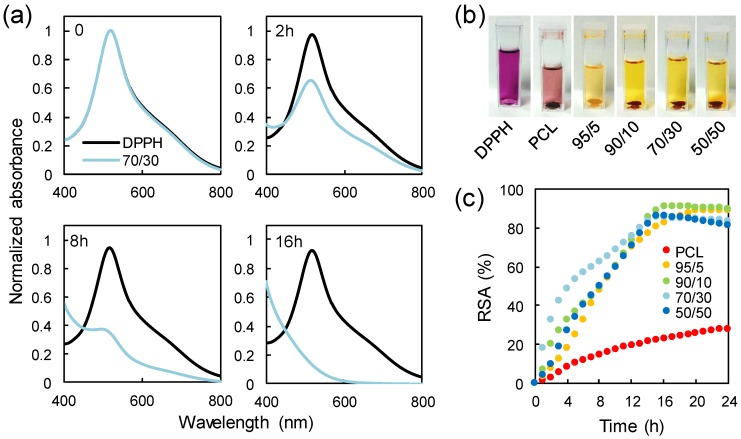
(**a**) Change in the absorption spectra upon reaction of 2,2-diphenyl-1-picrylhydrazyl (DPPH•) with a 70/30 sample. (**b**) Loss of the characteristic deep violet color of the DPPH• solution after being in contact with different samples for 24 h. (**c**) Radical scavenging activity of PCL and PCL/PFA blends tested against DPPH• in a spectrophotometric assay.

It is well known that several heterocyclic compounds possess antioxidant activity. For instance, coffee brews contain nearly 400 heterocyclic compounds [39], including furans, which not only give the characteristic roasted flavor to the beverages, but also are responsible for their antioxidant properties. Moreover, organic butter samples with high concentrations of furan fatty acids are known to be valuable antioxidants [40]. Finally, distilled spirits exposed to wood aging show antioxidant activity due to the presence of furans and phenols [41]. These aforementioned studies on the antioxidant effects of furan compounds naturally found in some dietary compounds or alcoholic beverages prompted us to test the radical scavenging activity of the PCL/PFA blend films. Radical scavenging activity was assessed by direct reaction of samples and DPPH• free radical as reported in a previous study [42]. DPPH• showed a strong absorption band close to 520 nm and the solution appeared deep violet. On accepting an electron or hydrogen from a donor, the solution lost its characteristic color and became pale yellow. As an example, Figure 6a displays the diminishing absorbance intensity of DPPH• upon its reaction with released compounds from a 70/30 film at different incubation times, as an example. Figure 6b shows the color change of the DPPH• solution from deep violet to pale yellow after being in contact with different samples for 24 h.

The resulting radical scavenging activity (RSA) for the neat PCL and the PCL/PFA blends is plotted as a function of the incubation time in Figure 6c. PCL exhibited a weak scavenging effect of ~28% after 24 h of incubation. The scavenging effect of PCL was probably due to the presence of residual oligomers and unreacted monomers in the polymer film along with its slight hydrolysis over time during storage or in solution [43,44]. Similarly, pure polylactic acid (PLA) demonstrated such low antioxidant activity, which was generally attributed to slight hydrolysis and residual lactic acid [45]. It was also shown that the PCL absorbed a certain amount of DDPH radicals during the experiments reducing absorption intensity of the radical species [46].

On the other hand, the presence of the PFA markedly increased the inhibitory effect towards DPPH•. In fact, a maximum radical inhibition of ~90% was reached after 16 h even with the addition of 5 wt % of PFA in the blend. As such, the blend 95/5 can act as an effective sustained antioxidant for PCL. As more PFA was added to the PCL/PFA blends, the contribution of the PFA to antioxidant performance (or reaction rate) after 16 h appeared negligible. However, it has to be noted that the reaction rate was not only related to the chemical properties of the samples, but also to mechanistic factors that may have altered the reaction mechanism [47,48]. For instance, DPPH• reaction rates were more closely related to concentration dependent steric accessibility than the chemical characteristics of test antioxidants. Specifically, as more PFA molecules were added to the system, random molecular packing may have impeded the access to DPPH•, blocking the required reaction at the radical site. Moreover, such impeding reactions may also be attributed to the increased viscosity due to higher PFA concentrations slowing the diffusion of antioxidants to the DPPH• radicals. The faster inhibition kinetics of 70/30 blends within the first 8 h could be attributed to such aspects.

### 3.7. Gas Barrier Properties

**Figure 7:**
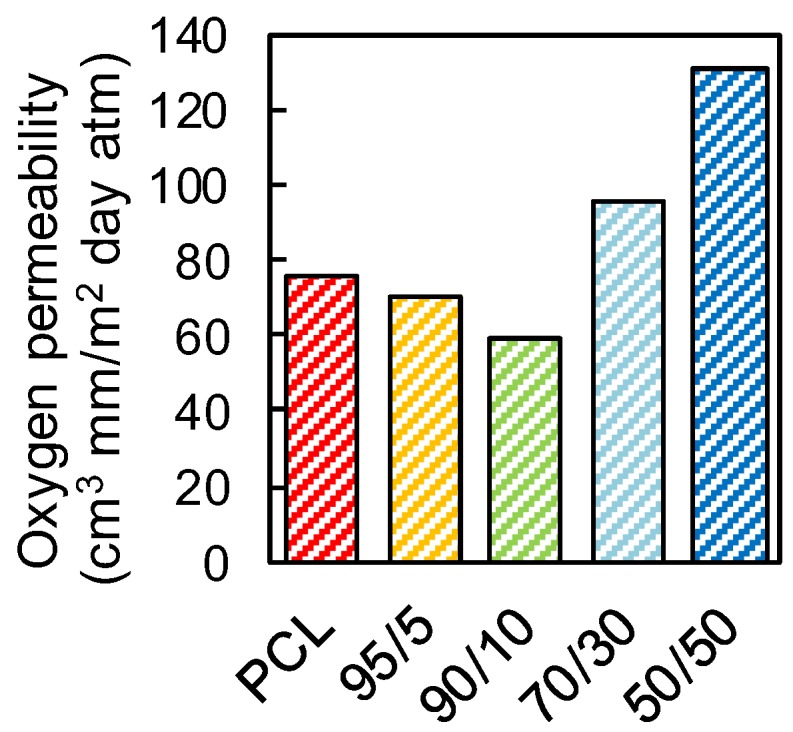
Oxygen transmission rate of neat PCL and PCL/PFA blends in 95/5, 90/10, 70/30 and 50/50 proportions.

Figure 7 shows the values of oxygen permeability through neat PCL and PCL/PFA blends. The PCL film showed a relatively high oxygen permeability of ~76 (cm^3^ mm)/(m^2^ day atm). The 95/5 and 90/10 films, however, showed lower permeability values of ~70 and ~60 (cm^3^ mm)/(m^2^ day atm), corresponding to reduction of ~6.6% and ~21% with respect to the neat PCL, respectively. The decrease in oxygen permeability may have been influenced by several factors, such as the ability of PFA molecules to fill the voids in PCL matrix, but also by scavenging oxygen molecules via chemical interactions between PFA and the oxygen (see Figure 6). At higher PFA concentrations exceeding 10 wt.%, the transmission rate increased. For 50/50 films, for instance, oxygen permeability levels were measured to be 73% higher than that of neat PCL. This could be due to the fact that PFA does not have the right polymeric structure (such as crystallinity) to form physical barriers for oxygen molecules even though the chemical scavenging properties were still active. It is well established, in fact, that the polymer structure influences the mass transport through it [49]. For example, crystalline regions have lower gas molecule diffusion rates than amorphous regions, which lack sufficient regularity in packing of the chains. Hence, we argue that due to high concentration of PFA in PCL, the rate of physical transport of molecular oxygen through the films was much faster than the chemical mechanisms that reduce oxygen molecules. 

For comparison purposes, the oxygen permeability values of various conventional polymers commonly used in packaging applications are shown in Table 1. The 90/10 blend, for instance, appeared to be as good as polypropylene (PP), polyethylene (PE) or polystyrene (PS) resins, all being 100% petroleum-based polymers. In a future work, various additives such as organoclays may be compounded to reduce the oxygen permeability values further.

## 4. Conclusions

In this study, PCL/PFA blend films were prepared at different PCL/PFA proportions by solution casting. AFM measurements revealed identical surface morphologies for all PCL/PFA blends and neat PCL films. ATR-FTIR spectroscopy analysis confirmed the absence of chemical interactions between PCL and PFA, while a decrease of the melting temperature enthalpy at increasing concentration of PFA in the blends was determined by DSC analysis, probably related to a loss of crystallinity, as confirmed by XRD measurements. Tensile test measurements showed that PFA at concentrations up to 20 wt % acts as plasticizer for PCL. As such, PFA was found to be compatible with PCL when blended and the resulting films demonstrate mechanical properties similar to plasticized PCL polymer. All PCL/PFA blends had good biocompatibility such that CHO cells proliferated up to complete confluence on substrates made of PCL/PFA. Additionally, the indirect toxicity tests showed that the same cells maintain their viability when growing in culture medium previously conditioned with PCL/PFA for as long as two days. Interestingly, scavenging activity tested against DPPH• revealed that PCL/PFA composite films possess potent antioxidant properties even at PFA content as low as 5 wt %. Finally, the oxygen barrier properties could be improved by adding up to 10 wt % of PFA into PCL/PFA blends. These results enable to design blends with tailored properties by selecting the appropriate composition for the development of a new class of cost-effective biomaterials with potential application in food packaging or biomedical devices. 

## Figures and Tables

**Table 1:** Permeability of polymers commonly used in packaging [49].

Polymer	Oxygen Permeability (cm^3^ mm)/(m^2^ day atm)
Poly(ethylene terephthalate) (PET)	1–5
Polypropylene (PP)	50–100
Polyethylene (PE)	50–200
Polystyrene (PS)	100–150
Poly(vinyl chloride) (PVC)	2–8
Poly(ethylene naphthalate) (PEN)	0.5
Polyamide (PA)	0.1–1 (dry)
Poly(vinyl alcohol) (PVAL)	0.02 (dry)
Ethylene vinyl alcohol (EVOH)	0.001–0.01 (dry)
Poly(vinylidene chloride) (PVDC)	0.01–0.3

## Supplementary Materials

The following are available online at https://www.mdpi.com/2073-4360/11/6/1069/s1, Figure S1: Extruded filaments of a 50/50 blend having different diameters, Figure S2: Optical view of CHO cells at the end of the indirect toxicity cells.
