# Supplementary material for: Poly(furfuryl alcohol)-Polycaprolactone Blends
Source: Polymers (Basel). 2019 Jun 20;11(6):1069. doi: 10.3390/polym11061069 (PMC6630956; doi:10.3390/polym11061069)
Supplement: Supplementary file 1 [file polymers-11-01069-s001.pdf]

Supporting Information

Poly(furfuryl alcohol)-Polycaprolactone Hybrids

Gabriele Nanni [1,*], José A. Heredia-Guerrero [1], Uttam C. Paul [1], Silvia Dante [2], Gianvito Caputo [3], Claudio Canale [4], Athanassia Athanassiou [1], Despina Fragouli [1] and Ilker S. Bayer [1,*]

[1] Smart Materials, Istituto Italiano di Tecnologia, via Morego 30, 16163 Genova, Italy; jose.heredia-guerrero@iit.it (J.A.H.G.); uttam.paul@iit.it (U.C.P.); athanassia.athanassiou@iit.it (A.A.)

[2] Nanoscopy & NIC@IIT, Istituto Italiano di Tecnologia, Via Morego 30, 16163 Genova, Italy; silvia.dante@iit.it (S.D.)

[3] Nanochemistry, Istituto Italiano di Tecnologia, Via Morego 30, 16163, Genova, Italy; gianvito.caputo@iit.it (G.C.)

[4] Department of Physics, University of Genoa, 16146 Genoa, Italy; canale@fisica.unige.it (C.C.)

* Correspondence: gabriele.nanni@iit.it; Tel.: +39-010-71781-352 (G.N.); ilker.bayer@iit.it; Tel.: +39-010-71781-566 (I.S.B.)

Extrusion of the blend

Filaments having different diameters (Fig. S1a) were obtained by extrusion and subsequent mechanical stretching. The extrusion was performed by feeding chopped films into a single screw extruder (Micro extruder, Rheoscam Scamex, France, Fig. S1b). The temperature along the extruder was 50-70°C and the speed of the screw was 30-40 rpm. The fibers were stretched manually immediately after extrusion.

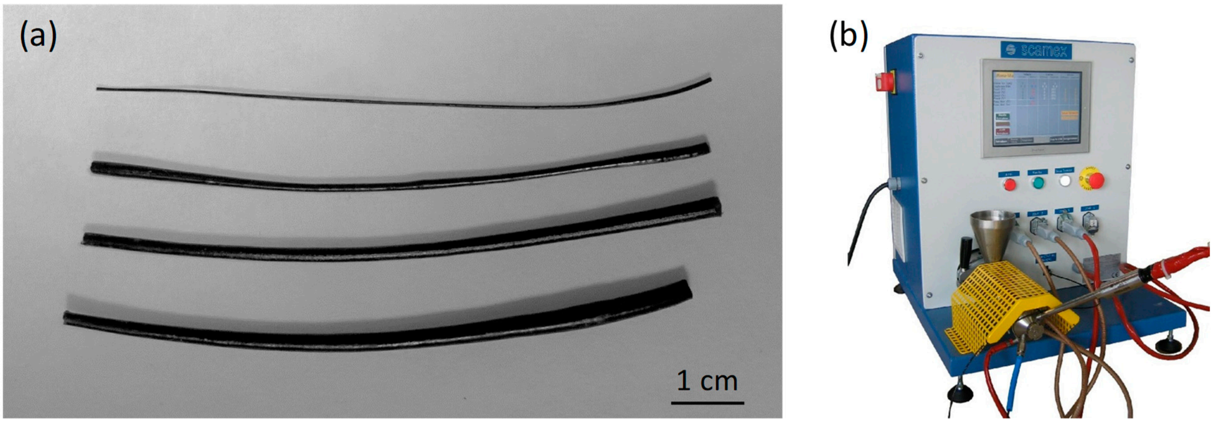

Figure S1. (**a**) Extruded filaments of a 50/50 blend having different diameters. (**b**) Extruder used to produce the filaments.

XRD patterns

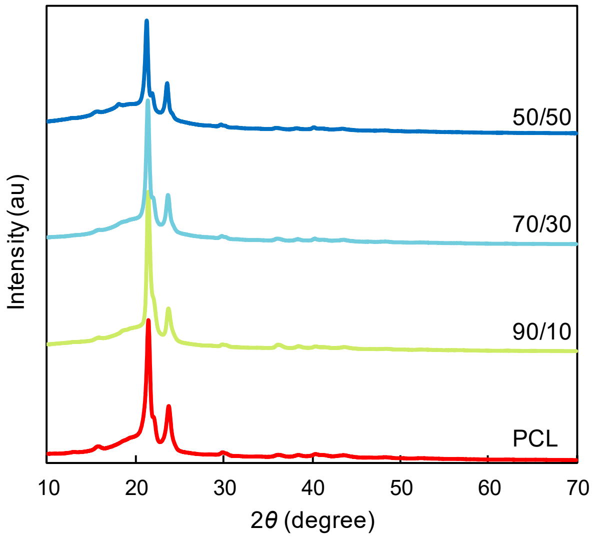

Figure S2. XRD pattern of PCL and different PCL/PFA blends.

Biocompatibility study

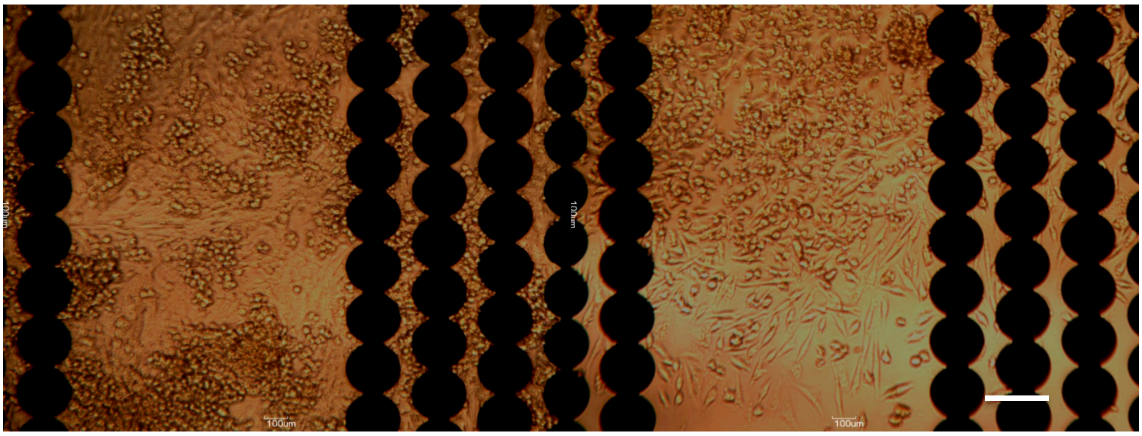

Figure S3. Optical view of CHO cells at the end of the indirect toxicity cells for control (left) and 50/50 sample (right). In both cases, cells were confluent; in the control CHO cells built multilayers and create aggregates (scale bar 100 µm).
